# Stimulus-Response Mappings Shape Inhibition Processes: A Combined EEG-fMRI Study of Contextual Stopping

**DOI:** 10.1371/journal.pone.0096159

**Published:** 2014-04-24

**Authors:** Christina F. Lavallee, Christoph S. Herrmann, Riklef Weerda, René J. Huster

**Affiliations:** 1 Experimental Psychology Lab, Psychology Department, European Medical School, Carl von Ossietzky University, Oldenburg, Germany; 2 Research Centre Neurosensory Science, Carl von Ossietzky University, Oldenburg, Germany; 3 Biological Psychology Lab, Psychology Department, European Medical School, Carl von Ossietzky University, Oldenburg, Germany; Ecole Polytechnique Federale de Lausanne, Switzerland

## Abstract

Humans are rarely faced with one simple task, but are typically confronted with complex stimulus constellations and varying stimulus-relevance in a given situation. Through modifying the prototypical stop-signal task and by combined recording and analysis of electroencephalography (EEG) and functional magnetic resonance imaging (fMRI), we studied the effects of stimulus relevance for the generation of a response or its inhibition. Stimulus response mappings were modified by contextual cues, indicating which of two different stimuli following a go stimulus was relevant for stopping. Overall, response inhibition, that is comparing successful stopping to a stop-signal against go-signal related processes, was associated with increased activity in right inferior and left midfrontal regions, as well as increased EEG delta and theta power; however, stimulus-response conditions in which the most infrequent stop-signal was relevant for inhibition, were associated with decreased activity in regions typically involved in response inhibition, as well as decreased activity in the delta and theta bands as compared to conditions wherein the relevant stop-signal frequency was higher. Behaviorally, this (aforementioned) condition, which demanded inhibition only from the most infrequent stimulus, was also associated with reduced reaction times and lower error rates. This pattern of results does not align with typical stimulus frequency-driven findings and suggests interplay between task relevance and stimulus frequency of the stop-signal. Moreover, with a multimodal EEG-fMRI analysis, we demonstrated significant parameterization for response inhibition with delta, theta and beta time-frequency values, which may be interpreted as reflecting conflict monitoring, evaluative and/or motor processes as suggested by previous work (Huster et al., 2013; Aron, 2011). Further multimodal results suggest a possible neurophysiological and behavioral benefit under conditions whereby the most infrequent stimulus demanded inhibition, indicating that the frequency of the stop-signal interacts with the current stimulus-response contingency. These results demonstrate that response inhibition is prone to influence from other cognitive functions, making it difficult to dissociate real inhibitory capabilities from the influence of moderating mechanisms.

## Introduction

Several cognitive functions allow humans the ability to flexibly adapt and change their behavior in a given situation. One of these cognitive control processes, commonly referred to as inhibition, allows us to stop our behavior when it is deemed inappropriate [1].Other cognitive functions include response-switching (a form of task-switching), which involves switching the response associated with a particular stimulus [2], [3]. In everyday situations, humans are often faced not only with merely inhibiting one response, but making a decision about how to respond to one or more stimuli, which may or may not be conflicting [4]. In these situations, some stimuli may be more or less relevant than others and there is often contextual information present which may change the speed and accuracy of performance or may shape advanced preparation of the upcoming process [5]. For example, in a typical traffic situation, traffic lights turn yellow before turning red in order to prepare the driver for an upcoming stop or to prepare drivers for the green light, which may change the driver’s current behavior, in that he would eventually reduce his acceleration in preparation for the red light or begin to prepare for acceleration, in the case that he expects a green light. Clinical populations, such as those with schizophrenia [6]–[9], attention-deficit hyperactivity disorder (ADHD) [10], [11], obsessive-compulsive disorder (OCD) [12], as well as others have problems with such inhibitory behavior. A popular paradigm utilized to study behavioral inhibition is the stop-signal task, in which one must override the dominant tendency to produce a response (i.e. responding to a go-signal) on go trials [13]. On stop trials, participants must withhold their response in reaction to infrequently presented stop-signals (i.e. exerting inhibition), which appear after the initial go-signal with a delay (usually termed the stop-signal delay or “SSD”). Conveniently, this paradigm may be modified by introducing contextual variations of stimulus-response mappings via changing the relevance and frequency of the “stop” stimuli, allowing us not only to discern effects of stop stimuli frequency, but also how additional task-irrelevant stimuli influence the stopping processes, if at all.

Theories on motor inhibition and response-switching via stimulus-response mapping have been developed separately and only few studies[14]–[16]have investigated how changing stimulus-response (S-R) mapping influences the inhibition process. Others have investigated the role of stop-signal relevance [17], how preparation influences the inhibition processes [18], [19], as well as probability effects in the stop-signal paradigm [20], [21].Probability, or frequency, effects in the stop-signal task have typically been studied with electroencephalography(EEG) methods and the functional magnetic resonance imaging (fMRI) literature for this particular topic is relatively sparse. Although both stop signal relevance and frequency seem to influence the inhibition processes, it remains to be determined whether these two key features interact and whether or not they exert an influence on stopping processes either at the behavioral or neurophysiological levels. A pressing question is whether changing S-R contingencies during a stop-signal task alters the neurocognitive mechanisms associated with stimulus processing as reflected in the spatio-temporal activation profiles of brain regions.

The relationship between S-R contingency variations and successful motor inhibition has not yet been thoroughly addressed in the literature; however, these interactions are important to discern based on the prevalence of response inhibition impairment in clinical disorders. Barch et al. stress a need for better characterization of response inhibition across neuropsychiatric disorders, as well as better delineation of different aspects of the stop-signal paradigm [6]. If, for example, inhibitory mechanisms interact with other cognitive control mechanisms enabling performance during S-R variations, and a clinical population demonstrates impairments in both domains, it is not easily possible to directly assess if both processes are impaired or if only one of the processes is disturbed, yet it indirectly causes deteriorated performance to the other. This could be one possible explanation for why cognitive impairments in ADHD and other syndromes seem so variable [22], [23]. Given the possibility of such interactions, the exact design of an experimental task becomes critical. Thus, in this study, we introduce a paradigm that allows us to test the interaction of S-R mappings and motor inhibition, giving us the opportunity to discern associated neuronal activation patterns. We also provide results demonstrating a possible brain/behavior benefit of stopping under certain conditions. These results certainly require further exploration with clinical populations to discern if the same pattern of response inhibition performance results persists.

Toward this end, we performed a simultaneous event-related EEG-fMRI experiment, in which we examined neural response patterns during successful motor inhibition under varying S-R contingencies. Stimulus-response mappings to stimuli were varied over three conditions within the stop-signal paradigm. These stimuli, not including the go-stimuli, were presented at different frequencies, and given the current stimulus-response contingency, the relevance of the stimuli would change, meaning that inhibition would be required if the given stimuli was “relevant” (i.e. relevant = stop; irrelevant = do not stop/continue pressing). By changing the relevance and frequency of the “stop” stimuli via stimulus-response mappings, we were able to test the influence of these factors on inhibition. In the spatial domain, response inhibition is mediated by the right inferior frontal cortex (rIFC) and the dorsomedial frontal cortex, especially the presupplementary motor cortex (preSMA), as well as the subthalamic nucleus (STN) [1]. A recent study, however, employing a modified stop signal paradigm has implicated the rIFC in a ventral attentional system and the preSMA in actual inhibition of the ongoing action [24], but it should be noted here that this idea has recently been challenged [25]. On the other hand, electrophysiological measurements reflect differences at a much higher temporal resolution [26].

A recent review [26] on the topic of electroencephalography of response inhibition found that the current role of oscillatory EEG responses in behavioral inhibition tasks is still rather ambiguous and requires further exploration; therefore, as opposed to a rather conventional ERP-based analysis. However, a common finding is increased theta and delta power during inhibition, assessed either via no-go or stop-trials during the 200 to 600 ms post-stop-stimulus time window [27]. Recently, intracranial recordings of patients have demonstrated inhibition-related increases in beta band activity [28], indicating another source of variance in the time-frequency domain possibly contributing to inhibitory performance. Nevertheless, as seen in the supplementary material, oftentimes features of time-frequency data may display spatiotemporal overlap and require further decomposition through means of an ICA to uncover source activity patterns otherwise hidden in the source-mixtures recorded at scalp EEG electrodes [29]–[31].

Simultaneous EEG and fMRI recordings and analyses were used to discern not only how cortical structures contribute to these processes (fMRI), but also how time-frequency characteristics of the EEG relate to such operations associated with contextual modulations of inhibition. Assuming a relative independence of neurocognitive mechanisms for the processing of stimulus frequency and stimulus-response mappings/task sets, we hypothesized that conditions with the highest number of relevant stop trials would elicit slower go reaction times (goRTs), as compared to conditions wherein the number of relevant stop trials was fewer [4], [19], [28], [32], [33]. Moreover, we predicted that trials with the fewest number of relevant stop signals would elicit more failed attempts at stopping (that is, a higher response rate; RR), given that the likelihood that a stopping mechanism is required is the most infrequent in this condition. Furthermore, one may expect higher EEG amplitudes in some frequency bands (i.e. theta) when stopping infrequently, as compared to more frequently. These general stimulus frequency effects would be due to well-known strategic adaptations in behavior (i.e. the speed-accuracy tradeoff); however, whether or not these well-known effects are stable during contextual stopping, with more than one stimulus type (relevant vs. irrelevant) and variations to S-R assignments over blocks of trials, remains to be discerned. By including such variations to stimulus frequency via altered stimulus-response mappings, one is able to assess stopping under more realistic conditions, as we are normally confronted with competing information in everyday situations. Moreover, the classic stop-signal paradigm is rather limited in this respect [4], stressing the need for more realistic stopping paradigms.

In short, behavioral results were not compatible with a purely frequency-driven explanation, thereby leading us to conclude that variations in the stimulus-response mapping, which change the relevance of the upcoming deviant stimuli, alter the inhibitory processes participants implemented when inhibiting a planned motor response. Correspondingly, such changes, when controlling for frequency of the relevant stop trials, were associated with relative decreases in activation in regions typically involved in response inhibition. This suggests that mechanisms associated with the processing of stimulus frequency and stimulus-response assignments interact and in conjunction shape behavioral inhibition, leading to increased neural efficiency under specific conditions.

## Materials and Methods

### Ethics Statement

All participants provided written informed consent prior to participating and the study was conducted in accordance with the principles expressed in the Declaration of Helsinki. The local ethics committee at the University of Oldenburg approved the protocol.

### Participants

Thirty-seven healthy participants were recruited from a database of subjects regularly participating in psychology/neuroscience experiments at the University of Oldenburg. All subjects (n = 37, 20 female, mean age = 23.8 years, standard deviation = 2.44 years) were right-handed according to the Edinburgh Handedness Inventory and none of the subjects had reported a history of psychiatric or neurological disorders and all had normal or corrected to normal vision. During the experimental session, participants performed a modified stop-signal task and simultaneous EEG-fMRI was recorded during the experiment. Datasets from 16 subjects were excluded due to technical malfunctions, an inability to properly remove MR-gradient artifacts from the EEG data or a failure to learn the task (n = 3), leaving an effective sample of 21 subjects (n = 21, 13 female, mean age = 24.46 years, standard deviation = 2.45 years).

### Experimental Design

**Figure 1:**
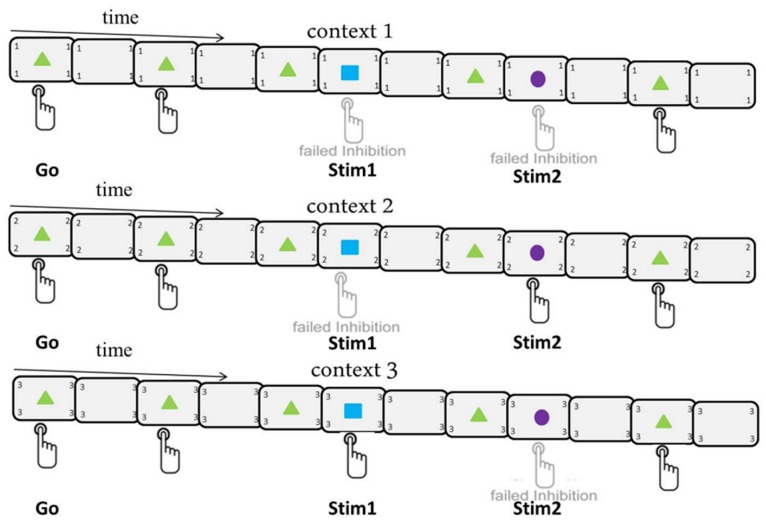
Experimental design. Depicted is the experimental design, outlining the three contexts and illustrating the response selection for the go signal (triangle) and two infrequently presented stimuli (square and circle). The current context is always presented in the four corners of the presentation screen. The presentation of Stim1 (13%) is more infrequent than the presentation of Stim2 (26%), whereas, the majority of trials are go-trials (61%). For context 1, a button press for Stim1 (blue square) and Stim2 (violet circle) is considered failed inhibition; whereas, a button press for either Stim1 (blue square) in context 2 or for Stim2 (violet circle) in context 3 is failed inhibition. That is, subjects are required to withhold their response to both Stim1 and Stim2 during context 1; however, in context 2 and context 3, stimulus-response mappings change such that the stopping demands are different (stop Stim1 in context 2 and Stim2 in context 3).

A modified stop-signal paradigm was implemented (See Figure 1), which was designed to test the interplay of varying stimulus-response mappings and motor inhibition. The stimuli in this visual stop-signal task were comprised of one go-signal and two infrequently presented “stop” stimuli (stimulus 1 and stimulus 2), which in a subset of trials succeeded the go-signal. Stimuli were comprised of forms square, triangle, and circle, on the one hand and colors blue, violet, and green on the other hand. The assignment of form and color to the stop- and go-signals were fixed for a given subject but counterbalanced across subjects. Go-signals and infrequently presented stimuli (i.e. stimulus 1 and stimulus 2) were presented for 100 ms. Sixty-one percent of all trials were pure go trials, 13% of trials consisted of stimulus1 (Stim1) being presented after a go-signal and 26% of trials consisted of stimulus 2 (Stim2) being presented after a go-signal. Subjects were not given information about the specific proportion of go and stop trials; however, they were given instructions on how to deal with these infrequently presented stimuli and these instructions differed based on the current stimulus-response mapping contexts of the experiment. In essence, three stimulus-response contexts were present in the experiment and the subject was constantly reminded of the context in the form of a number (1, 2 or 3) being presented in the four corners of the presentation screen. Within Context1, the subjects were instructed to stop their response in reaction to the appearance of both Stim1 and Stim2, which is most reflective of the classic stop-signal task as participants must withhold their response to all infrequently presented stimuli. For Context2, participants were instructed to withhold their response in reaction to Stim1 and continue to respond when Stim2 was presented; whereas, the instructions were reversed (Stim1: respond; Stim2: withhold) for Context3 (See Figure 1).

This design allowed us to make specific contrasts to directly test the effects of behavioral relevance of deviating stimuli. Across contexts the stimuli and their corresponding quantities were kept constant, yet the frequency of task-relevant stop stimuli varied across contexts. Within Context1, for example, all infrequently presented stimuli (39%) are behaviorally relevant and can be considered stop signals; however, in context 2 and 3 only subsets of the infrequently presented deviating stimuli (Stim1 in Context 2 (13%) and Stim2 in Context3 (26%)) were behaviorally relevant. Hence, comparing context1 to context2, subjects would be expected to inhibit their responses to all deviant stimuli in the first context, but only to inhibit their responses to the deviant stimuli presented after 13% of go-trials in context 2, while continuing to respond to the other 26% of (stop irrelevant) stimuli. Likewise, in the third context, subjects would be expected to inhibit their response to the deviant stimuli presented on 26% of trials and would be expected to continue responding to the remaining 13% of deviant stimuli. A more detailed description of the statistical analysis is given below.

Overall, subjects had to process a total of 1152 trials of which 708 were go (61%) and 296 were stop trials (39%) and each trial had a 1350 ms response active window. The remaining 148 trials involved infrequently presented stimuli and were fundamentally go trials where stim1 (13%) or stim2 (26%) did not require inhibition (Context2 and Context3, respectively). In total, 384 trials were processed for each context, consisting of 236 go-trials per context, 148 stop-relevant trials for Context1, 48-stop relevant trials for Context2 (100 stop-irrelevant trials) and 100 stop-relevant trials for Context3 (48 stop-irrelevant trials). Between 13 and 18 trials comprising the same context (that is, either 1,2,3) were presented consecutively and the presentation of these trials across contexts was pseudo-randomized, whereby the overall amount of trials was the same for each of the three contexts. All responses were made via a button-press with the right index finger. The stimulus-onset asynchrony (SOA) between the go-signal and successive stim1 or stim2 stimuli was dynamically adjusted based on the error rate of the stop trials: the initial delay of 128 ms could lead to an increase of 16 ms if inhibition was successful, or a decrease of 64 ms should inhibition be unsuccessful. SOA upper and lower boundaries were set at 928 ms and 64 ms, respectively. This tracking procedure was adjusted dynamically to yield an approximate average error rate of 20% [34] to ensure a sufficient number of successful stop trials for analysis. Between consecutive trials, screens with a central fixation cross (not depicted in Figure 1) and numbers in the four corners indicating the context of the upcoming trial were presented. Trial onsets were randomly jittered between 0 and 2000 ms relative to the acquisition of fMRI volumes to allow for an increased temporal sampling of the hemodynamic response (HR). Thus, a trial was comprised of a go signal presented after a randomly chosen jitter (between 0 and 2000 ms), during which time a central fixation cross was displayed. The whole experimental session, which lasted 42 minutes, was preceded by a practice session (10 mins) outside the scanner. Throughout the experiment, subjects received context-wise feedback in the form of written text for 2 s after each 13–18 trial consecutive presentation (mentioned above) instructing them to adjust their responses based on accuracy and reaction time. During the experiment, subjects had a 2 minute pause every 288 trials (9.6 mins) where the instructions were shown on the presentation screen and the scanner was left running. Following the experimental session, T1-weighted structural images were obtained from all subjects to aid in the preprocessing of functional images.

### fMRI Data Acquisition and Preprocessing

Functional and structural images were acquired with a 1.5 Tesla MRI scanner (Siemens MAGNETOM Sonata, Siemens AG, Erlangen, Germany). Functional images were obtained using a multislice T_2_* weighted echo planar imaging (EPI) sequence (TE = 50 ms, TR = 2000 ms, flip angle 90°, 25 axial slices in ascending order, slice thickness 3 mm with a 10% distance factor, FOV 200×200 mm) resulting in a voxel size of 3.1×3.1×3.0 mm^3^. During the experiment, 1293 whole brain scans (42 minutes) were acquired. Structural T1-weighted images were obtained after the experiment (voxel size 1 mm isotropic, 176 slices, FOV = 256×256 mm^2^, TR = 2130 ms, TE = 3.93 ms and 15° flip angle). The preprocessing of the images was completed using the SPM8 software package (FIL, Wellcome Trust Centre for Neuroimaging, UCL, London, UK: http://www.fil.ion.ucl.ac.uk/spm) running under MATLAB. Spatial realignment was performed to adjust for movement-related shifts across volumes over time and a temporal correction for the differences in acquisition time between slices of the collected volumes was performed (slice time correction). All images were then normalized to the MNI (Montreal Neurological Institute, Quebec, Canada) EPI-template provided by SPM. Lastly, functional images were smoothed with a three-dimensional Gaussian kernel of 8 mm full-width-at-half-maximum (FWHM).

### EEG Recording and Preprocessing

In addition to fMRI data acquisition, the EEG was simultaneously recorded by an MR-compatible amplifier (BrainAmp MR Plus, Brain Products) that was especially designed to meet not only safety requirements but also to optimize signal quality when collecting simultaneous EEG and fMRI data. EEG data was obtained from 64 electrodes placed in accordance with the 10-10 system for electrode placement. EOG electrodes recorded eye artifacts and an electrode placed to the left of the spinal column between the 5^th^ and 7^th^ costa monitored electrocardiogram activity. A commercial device (Sync Box, Brain Products) was utilized to synchronize the hardware clock of the EEG with the MRI scanner’s gradient switching system. FCz served as online reference to keep the distance between recording reference and “active” electrodes small, thereby minimizing the chance of amplifier saturation. The data was recorded with a passband of 0.016–250 Hz, digitized at 5000 Hz and 16 bit with 0.5 µV resolution (dynamic range, 16.38 mV). EEG data was corrected for MR gradient and ballistocardiac artifacts by applying modified versions of the algorithms proposed by Allen and colleagues [35], [36]) as implemented in EEGLAB [37]. The MR-denoised EEG data were low-pass filtered at 35 Hz, re-referenced to the common average reference and down-sampled to 250 Hz. In order to enhance the signal to noise ratio, and to allow for single-trial analyses, the data were decomposed by means of a temporal independent component analysis (extended infomax) and components representing muscle, eye or residual cardioballistic artifacts were removed.

### EEG Parameterization

Time-frequency decompositions were calculated using functions provided by the EEGLAB open source software [37]. Frequencies from 0 up to 35 Hz were analyzed utilizing 100 frequency steps. The upper and lower boundaries for the baseline correction were −800 and −100 ms, respectively, before stimulus onset. Power values for each time-frequency bin from stimulus onset (0 ms) up until 1214 ms post-stimulus onset were normalized by dividing by the frequency-specific power during baseline. A subsequent log-transformation was computed on data from all electrode positions to acquire dB time-frequency values (event-related spectral perturbations, ERSP [37], [38]). Then, mean ERSP-values from electrode-sites of interest (EOIs) within temporally specific windows for different frequency bands were extracted. Delta (0–4 Hz) time-frequency values were extracted between 300 and 400 ms post stimulus-onset; whereas, theta (4–8 Hz) values were extracted between 150 and 250 ms post stimulus-onset. These time ranges were chosen to correspond with the typical temporal progression of the N2-P3 complex observed in stop-signal paradigms [26]. The close correspondence between N2 and theta, as well as P3 and delta in inhibition paradigms [27] has resulted in an interpretation wherein theta activity in the N2 time-window may correspond to conflict-related effects [20]; whereas, delta and the P3 may be associated with response-related evaluative processing stages and the evaluation of motor inhibition as opposed to directly reflecting motor inhibition [26]. Low-beta (12–21 Hz) and high-beta (21–30 Hz) values were extracted between 220 and 500 ms post stimulus-onset, corresponding to reports of Swann et al [28], [39]and Ritter et al [40]. These time-frequency features were extracted for defined EOIs, which were created by calculating mean activity over the single electrodes, as specified below. Anterior (frontal) and posterior (parietal and/or motor), as well as central and lateral (left/right) EOIS were used for subsequent analyses. These EOIs included frontocentral (F1, F2, FC1, FC2, FCz, Cz) and centroparietal (C1, C2, CP1, CP2, CPz, Pz) regions, corresponding to sites of increased delta and theta band activity during stop-signal tasks [26]. Frontal and motor cortex regions, including the left (F7, F5, AF3, AF7) and right (F6, F4, AF4, AF8) inferior frontal cortices and the bilateral motor cortices (Left: C5, C3, CP3; Right: C4, C6, CP4) were also identified, as we hypothesized beta activity to be associated with these regions, as others [28] have previously identified in inhibition studies. By extracting these specific ERSP values, we are able to discern how changes in stimulus-response mapping affect time-frequency characteristics typically observed during the stop-signal task.

### Statistical Assessment

Behavioral features of the stop-signal task, such as go reaction time (go-RT), response rate (percentage of unsuccessful stop trials) and stop-signal reaction time (SSRT) were analyzed with repeated measures ANOVA (with CONTEXT, referring to *stimulus-response context*, as within-subject factor). Behavioral results, when in accordance with hypotheses, were also used to guide contrasts for later EEG, fMRI and multimodal analyses. The response rate measure is computed as the number of failed stop trials divided by the number of all stop trials and can be considered a measure of stopping accuracy, indicating that, the lower the number, the more successful participants were on stop trials. One measure unique to the stop-signal task is the SSRT, which is based on the horse-race model of stopping. This model describes the stopping and going processes as competing for the first finishing time and has been utilized to describe stop-signal task performance (Logan, Cowan & Davis, 1984). Because the stopping mechanism itself cannot be directly measured, the SSRT has been proposed to represent an estimate of the time required for stopping the response. Estimation of the SSRT can be calculated utilizing numerous methods [34]; however, recent research has proposed utilizing the integration method [41], as this method may represent a more robust measure, especially given go-trial related strategies [42]. With this method, one assumes that the finishing time of the stop process corresponds to the goRT at position *n,* where n is equal to the number of RTs in the distribution multiplied by the overall response rate (p(respond|stop signal)). The SSRT is then estimated by subtracting the mean SOA from the goRT at position *n.* [42].

**Figure 2:**
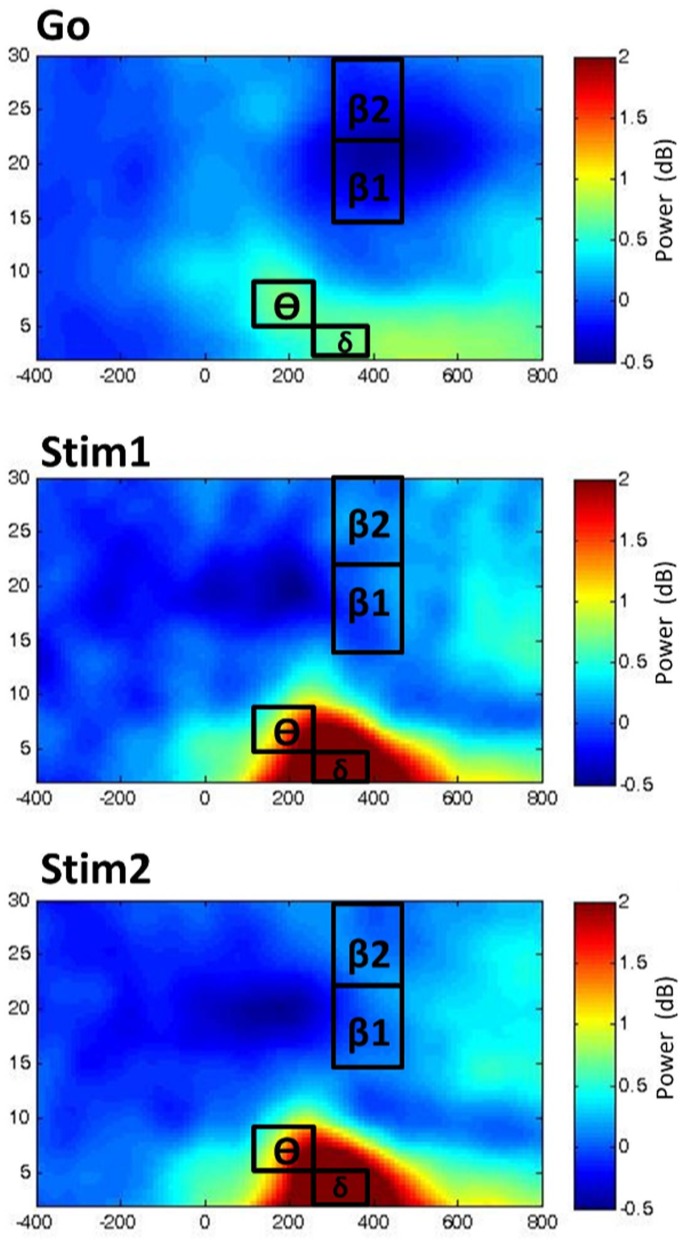
Time-frequency plots. The time-frequency plots for Go, Stim1 and Stim2 for context1 from electrode FCz are displayed in power (dB) from 400 ms prior to stimulus onset (0) to 800 ms post-stimulus onset. The temporal windows and associated time-frequency bands δ = delta, θ = theta, β1 = low beta, β2 =  high beta) are illustrated directly on the plots.

Unimodal time-frequency EEG data was analyzed with the use of repeated measures ANOVAs, with factors CONTEXT (1,2,3), STIMULI (Go, Stim1, Stim2), REGION (anterior, posterior), as well as LATERALITY (left, middle, right) as within-subjects factors in order to discern regional changes in time-frequency data in relation to the stimuli-type and context. For the unimodal EEG analysis of the time-frequency data, clusters of electrode channels were averaged and treated as a site of interest, as clarified in the *EEG Parameterization* section. Time-frequency plots are shown for the three stimuli in Context1, where the respective time-windows and EEG-frequency bands are illustrated on the plots (Figure 2).

Response inhibition, only when successful, was assessed by contrasting the go and Stim1 stimuli in Context1 (Stim1-Context1 vs. Go-Context1), as this is the type of contrast most often utilized in stop-signal studies to discern properties of response inhibition. That is, behavioral inhibition following presentation of the infrequent stop-signal as compared to that of the prepotent response-tendency associated with go-trials. We were then able to discern properties of response inhibition under varying stimulus-response contingencies by comparing responses to the equivalent deviant stimulus across contexts, while simultaneously controlling for frequency related effects. That is, Stim1 appeared following a go-signal on 13% of all trials in all contexts, but was relevant for stopping only in Context1 and Context2.On the other hand, Stim2 appeared following a go-signal on 26% of all trials in all contexts, but was relevant for stopping only in Context1 and Context3. Under conditions where the deviant stimuli (that is, Stim1 in Context3 or Stim2 in Context2) were not behaviorally relevant for stopping, subjects were required to respond as if it were a go-trial. The contrasts to assess effects of varying stimulus-response contexts (i.e. Stim1-Context1 vs. Stim1-Context2, as well as Stim2-Context1 vs. Stim2-Context3) were identified and focused on. Thus, we were able to directly compare relevant contextual changes in S-R contingencies for inhibition while still controlling for frequency of stop-signal stimuli. Statistical analyses were completed with Statistica (StatSoft).

The unimodal fMRI analysis was completed in SPM8 by first conducting a single-subject (first-level) analysis using a 128 s high-pass filter, utilizing the canonical HRF as a basis function. Six movement regressors calculated during realignment were entered and autoregressive modeling was utilized to account for serial c orrelations in the fMRI time series. Contrast images, including one contrast for each context and stimuli combination, as well as false alarms (i.e. commission errors) were subsequently entered into the group (second-level) analysis. Although no further analysis was completed with the false alarms, as we were only interested in successful response inhibition, these events were modeled to account for additional sources of variance. At the group level, a flexible factorial model was created with factors CONTEXT (1,2,3) and STIMULI (Go, Stim1, Stim2) as well as the reserved SUBJECT factor implicit in SPM8. Regional differences for variations in S-R contingencies (i.e. Stim1-Context1 vs. Stim1-Context2 and Stim2-Context1 and Stim2-Context3) and reactive response inhibition (Stim1-Context1 vs. Go-Context1) were explored via repeated measures t-tests (whole brain analysis, height threshold: p<0.05, corrected for multiple comparisons by family-wise error (FWE) correction on the voxel level; extent threshold: 10 voxels; same thresholds were applied to all other contrasts), in both directions (i.e. increases and decreases). Planned comparisons in the form of repeated measures t-tests in the context of a flexible factorial model were chosen as opposed to systematically discerning main effects and interactions, as, firstly, we were only interested in specific contrasts across S-R mappings, defined by our hypotheses and the behavioral findings and, secondly, such an analysis completed on the behavioral and unimodal EEG data guided subsequent uni- and multimodal fMRI contrasts, allowing us to focus on specific effects rather than employing an omnibus approach.

Those EEG frequency bands which were able to significantly differentiate stopping across S-R contexts for the aforementioned contrasts were utilized in the single-trial EEG-parameterization (the so-called EEG-informed fMRI analysis scheme) [43].These time-frequency features included frontocentral delta and theta, as well as left motor high beta extracted from the same time windows detailed earlier. The delta, theta and high beta parameters were modeled together at the single-subject level in one design. Here, parameterization of the hemodynamic response function (HRF) for multimodal analysis is accomplished such that modeled signal changes following events with a large EEG response are scaled up as compared with trials that demonstrate a smaller EEG response. Group-level analysis consisted of creating flexible factorial models for each of the three time-frequency features with CONTEXT (1,2,3) and STIMULI (Go, Stim1, Stim2) and the implicit SPM factor SUBJECT as factors. For the multimodal analysis, contrast images from each of the context and stimuli combinations were entered in to the analyses, consistent with the unimodal fMRI analysis.

## Results

### Behavioral Results

Means and corresponding standard error of the mean are reported for all behavioral measures (that is, go-RT, SSRT and RR) across all three contexts in Table 1. Go-RTs differed as a result of the context (F(2,20) = 6.25, p<0.005, η^2^ = .2389). Post-hoc testing using Tukey’s Honestly Significant Difference (Tukey HSD) revealed that go-RTs for Context2 (M = 644.43 ms, SEM = 23.83 ms) were significantly lower when compared to Context1 (M = 669.19 ms, SEM = 27.02 ms; p<0.0001), as well as compared to Context3 (M = 658.57 ms, SEM = 25.65 ms; p<0.005), suggesting faster responses to go-signals under S-R contingencies whereby the most infrequent stimuli required inhibition. Furthermore, reaction times on unsuccessful stop-trials (that is, when subjects failed to inhibit their response to a stop-signal) were significantly lower (F(1,19) = 79.74, p<0.0001, η^2^ = .808) when compared to go reaction times. Faster reaction times on unsuccessful stop trials (Context1: M = 499.7, SEM = 12.513; Context2: M = 509.4, SEM = 31.65, Context3: M = 516.29, SEM = 23.53), as compared to go trials are in line with the assumptions of the horse-race model of stopping and going processes [13].

**Table 1:** Behavioral Data.

Measure	Context1 (39%)	Context2 (13%)	Context3 (26%)
GoRT	669.19 (27.02)	644.43 (23.83)	658.57 (25.65)
SSRT	197.99(31.173)	250.129 (39.61)	224.88 (34.06)
RR	0.187(0.011)	0.166 (0.0185)	0.204 (0.014)

Under Context2 and 3, stim2 and stim1 were, respectively, not relevant for stopping and thus required the subjects to continue responding. The accuracy of responding under these conditions was not significantly different from normal go-trials, nor were the conditions significantly different from each other (Stim2-Context2: M = 0.949, SEM = 0.01; Stim1-Context3: M = 0.950, SEM = 0.01). Reaction times to these stop-irrelevant stimuli (that is, Stim2 in Context2 and Stim1 in Context3) demonstrated response slowing as compared to goRTs (p<0.05) on go-trials. Context-wise differences (F(1,20) = 6.225, p<0.05, η^2^ = .237) were also observed, indicating that the condition with fewer stimuli demanding a response (that is, Stim1-Context3) demonstrates longer reaction times (M = 891.238, SEM = 39.61) as compared to the condition with comparatively more stimuli (that is, Stim2 in Context2: M = 850.43; SEM = 41.15).

Typically, studies report faster go-RTs to be associated with less frequent stop signals, and these effects are, consequently, normally associated with increased errors [21], [44], [45]; this, however, was not the case in our study. Although response rates across the three contexts differed (F(2,20) = 5.026, p<0.05, η^2^ = 0.201), post-hoc testing using Tukey’s HSD revealed that response rates were lower for Context2 (M = 0.1658, SEM = 0.0185) as compared to Context3 (M = 0.204, SEM = 0.014; p<0.05) and Context1 (M = 0.187, SEM = 0.011; p<0.05). Response rates for Context3 also differed significantly (p<0.05). The time between the presentation of go and stop-signals (i.e. the SSD) were also fastest under Context2 (M = 273.57, SEM = 22.49) as compared to Context1 (M = 381.33 SEM = 28.16) and Context 3(M = 333.47, SEM = 27.44), as one would expect based on response rates (F(2,19) = 33.02, p<0.0001, η^2^ = .776). Thus, variations found regarding RTs and response rates cannot simply be explained in terms of a speed-accuracy tradeoff, which would typically be observed only when the stop stimulus frequency is varied. Thereby, this pattern deviates from results one may expect based purely on an effect of stop signal frequency. Additionally, results from a repeated measures ANOVA did not yield significant differences among SSRTs across contexts (F(2,19) = 0.76, p>0.4). The mean values can be found in Table 1.

Contrasts for the EEG and fMRI analyses were adapted to these behavioral results. As the pattern of behavioral results including reduced error rates and go-RTs suggests a behavioral benefit of S-R mappings as employed in Context2 as compared to Context1, further analyses were conducted to capture the neurophysiological processes contributing of these effects.

### Unimodal EEG Results

**Figure 3:**
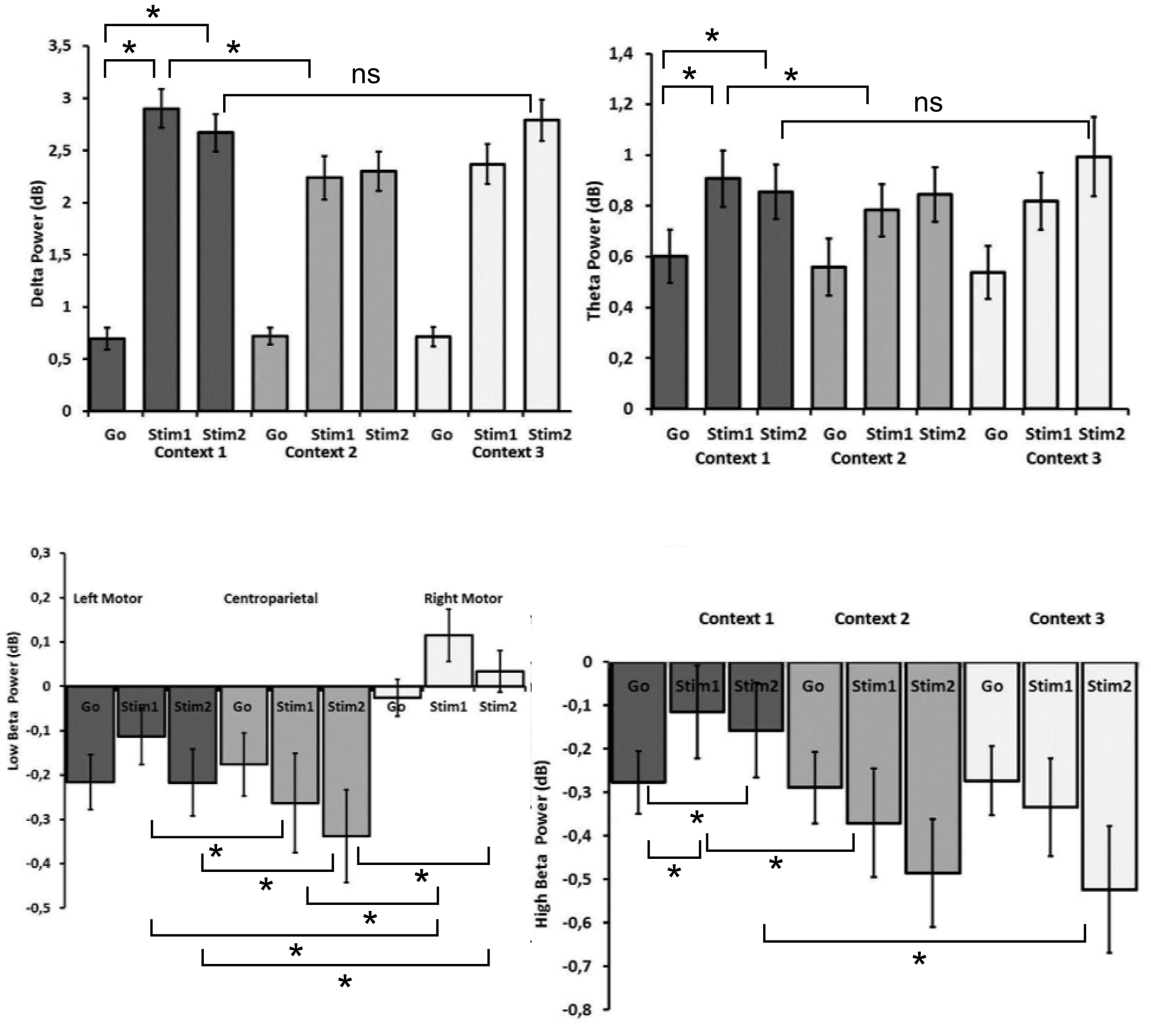
Effects and planned comparisons of time-frequency data. Mean power values and corresponding standard error of the mean (SEM) are shown for frontocentral delta, frontocentral theta, left motor high beta and low beta band activity across stimuli and contexts where the given interactions, as discerned by repeated measures ANOVA were significant. Where appropriate, planned comparisons are indicated (a star (*) indicates p<0.05, NS means “not significant”). Note that theta and delta power values are higher for Stim1 and Stim2 as compared to go-stimulus power, where significant differences across contexts for these stimuli are modulated by stimulus-response conditions requiring most infrequent inhibition control. In contrast, high beta power reductions are most prominent for the go-stimulus power in context 1; whereas, regional reductions in low beta power are most prominent over left motor and centroparietal regions, as compared to the right motor cortex.

Unimodal EEG analyses were conducted to discern significant changes in the specified time-frequency bins across previously defined electrode sites of interest for the stimulus-types and contexts via repeated measures ANOVAs (please refer to Figure 3).

### Delta

Results from a repeated measures ANOVA, displayed in Figure 3, yielded a significant three-way interaction between LATERALITY, STIMULI and CONTEXT (F(8,160) = 2.71, adjusted p<0.05, η^2^ = 0.12, Epsilon = 0.52). Planned comparisons and post-hoc tests were used to disentangle effects driving this three-way interaction. Overall, effects were most pronounced at midline compared to other electrodes with both Stim1 (frontocentral: M = 2.9, SE = 0.18; F(1,20) = 180.36, p<0.0001; centroparietal: M = 2.39, SE = .79; F(1,20) = 174.38, p<0.0001) and Stim2 (frontocentral: M = 2.67, SE = 0.18; F(1,20) = 140.87, p<0.0001; centroparietal: M = 2.15, SE = 0.17, F(1,20) = 94.01, p<0.0001) evoking higher amplitudes than go-signals (frontocentral: 0.69, SE = 0.102, centroparietal: M = 0.70, SE = 0.104; ) in Context1, thereby showing the expected difference associated with response inhibition. Delta power values across the three contexts can be viewed for the frontocentral EOI in Figure 3 (top left). Further planned comparisons showed decreased amplitudes over the frontocentralEOI in response to Stim1 when comparing Context2 (M = 2.38, SE = 0.21; F(1,20) = 10.83, p<0.005) to Context1 (M = 2.9, SE = 0.18), thereby suggesting a modulatory effect of varying S-R contingencies, while still controlling for how often the relevant stop signal appears. When contrasting Stim2-Context1 with Stim2-Context3, however, which is another possibility to test for variations in S-R mappings while controlling for frequency of the relevant stop signal, frontocentral activity was not modulated under these conditions. Taken together, these effects indicated increased regional delta activity during response inhibition; however, during Context2 containing the least frequently presented relevant stop stimuli, a relative decrease of delta activity (lower increase of activity in response to stop stimuli) is observed as compared to when S-R mappings indicate that all stop signals are relevant (Context1).

### Theta

A main effect of STIMULI (F(2,40) = 15.38, adj. p<0.001, η^2^ = 0.44, Epsilon = 0.764) indicated that theta power for both Stim1 (M = 0.92, SE = 0.086, Tukey HSD p<0.001) and Stim2 (M = 0.85, SE = 0.076, Tukey HSD p<0.001) was greater than for go-signals (M = 0.56, SE = 0.065). Furthermore, planned comparisons, as can be seen in Figure 3 (top right) revealed increased frontocentral theta activity in Context1 for Stim1 (M = 0.91, SE = 0.11, F(1,20) = 9.2, p<0.01) and Stim2 (M = 0.86, SE = 0.12; F(1,20) = 5.83, p<0.05), compared to go stimuli (frontocentral: M = 0.60, SE = 0.10), demonstrating the expected response inhibition effect, similar to that observed for delta activity. A modulatory effect of S-R mapping was observed in the frontocentral region with planned contrasts when comparing Stim1 from Context2 (M = 1.07, SE = 0.107) and Context1 (M = 1.39, SE = 0.116), whereby lower theta activity was observed when S-R contingencies required inhibition to the most infrequent relevant stop signal (F(1,20) = 5.01, p<0.05). For theta band activity, one would likely expect higher power to be associated with the less frequently presented stop stimuli, given the relationship between theta and N2-amplitude with conflict [26]; however, our results demonstrated the opposite, suggesting that we are not able to attribute effects only to stop stimuli frequency. The second contrast for variations in S-R mappings, Stim2-Context1 versus Stim2-Context3, did not reach significance, indicating that processes contributing to response inhibition were only modulated with the most infrequent relevant stop signal.

### Low Beta

A three-way interaction, depicted in Figure 3 (bottom left) involving the factors REGION, LATERALITY and STIMULI (F(4,80) = 8.38, adj. p<0.0001, η^2^ = 0.29,Epsilon = 0.75 ) revealed reduced low beta values in frontocentral and centroparietal EOIs for Stim1 (M = −0.26, SE = 0.11, Tukey’s HSD p<0.0001) when compared to the go-stimuli (M = −0.176, SE = 0.07). Further, post-hoc testing revealed that left (Stim1: M = −0.11, SE = 0.06, Tukey’s HSD p<0.005; Stim2 = 00.22, SE = 0.07, Tukey’s HSD p<0.0001) and right (Stim1: M = 0.11, SE = 0.59, Tukey’s HSD p<0.0001; Stim2: M = 0.034, SE = 0.045, Tukey’s HSD p<0.005) low beta over the motor cortices was higher than centroparietal low beta for Stim1 (M = −0.26, SE = 0.11) and Stim2 (M = −0.33, SE = 0.105).

Furthermore, planned comparisons revealed an increase in low beta activity for the left and right motor cortices during response inhibition in Context1 for Stim1 (left motor: M = −.055, SE = 0.073; F(1,20) = 9.04, p<0.01; right motor: M = .147, SE = 0.073; F(1,20) = 5.49, p<0.05) and Stim2 (left motor: M = −0.044, SE = 0.066; (F(1,20) = 8.67, p<0.01; right motor: M = 0.13, SE = 0.051; F(1,20) = 7.73, p<0.05 ) as compared to go trials (left motor: M = −.244, SE = 0.06, right motor: −0.016, SE = 0.041); however, low beta amplitudes did not vary across frontal EOIs nor during variations to S-R mappings. Please refer to Figure S3 in File S1 for a graph of means across stimulus-response contexts. This indicates that low beta activity was only affected by response inhibition predominantly over motor regions and that the particular S-R contingency did not modulate these effects.

### High Beta

A two-way interaction involving the factors REGION and STIMULI was observed (F(2,40) = 12.7, adjusted p<0.001, η^2^ = 0.39, Epsilon = 0.74), where post-hoc testing revealed that high beta values at anterior regions were significantly higher for Stim1 (M = −.0015, SE = 0.076, Tukey’s HSD p<0.005) and Stim2 (M = −0.065, SE = 0.077, Tukey’s HSD p<0.001) as compared to posterior regions (Stim1: M = −0.11, SE = −0.078; Stim2: M = −0.2, SE = 0.08 ); whereas, high beta between the anterior and posterior regions was not significantly different during go trials. Planned comparisons, as seen in Figure 3 (bottom right) revealed increased bilateral frontal high beta activity in Context1 for Stim1 (left frontal: M = 0.072, SE = 0.09; F(1,20) = 13.56, p<0.005; right frontal M = 0.025, SE = 0.08; F(1,20) = 7.35, p<0.05) and Stim2 (left frontal: M = 0.032, SE = 0.095; F(1,20) = 6.98, p<0.05; right frontal: M = 0.054, SE = 0.083; F(1,20) = 12.48, p<0.005) as compared to high beta activity during go-signal trials of Context1 (left frontal: M = −.136, SE = 0.062, right frontal: M = −.122, SE = 0.051). However, under conditions of altered S-R mappings where the least frequent stop signal is relevant, a decrease in left (F(1,20) = 12.41, p<0.005) and right (F(1,20) = 4.44, p<0.05) high beta over the motor cortices was observed for Stim1-Context2 (left motor: M = −0.369, SE = .124; right motor: M = −.0726, SE = 0.11), as compared to Stim1-Context1 (left motor: M = −0.11, SE = 0.106; right motor: M = 0.071, SE = 0.087), wherein(in Context1) all stop signals were relevant (See Figure 3, bottom left). The second contrast to discern variations in S-R mappings (i.e. Stim2-Context1 versus Stim2-Context3) was significant for the left motor EOI (F(1,20) = 4.45, p<0.05), and indicated decreased high beta power when only a subset of stimuli was relevant for stopping (Stim2-Context3: M = −0.334, SE = 0.112) as compared to conditions when all stop stimuli were relevant (Stim2-Context1: M = −0.115, SE = 0.107). These results indicate that response inhibition was associated with less negative amplitudes in the high beta frequency range, and that changing the relevance of the stimuli via altered S-R mappings resulted in attenuated high beta activity as compared to conditions where all stop stimuli were relevant.

Taking into account the aforementioned results, planned comparisons for the uni- and multimodal fMRI data were conducted to capture the effects observed in behavioral (goRT, RR) and EEG (delta, theta, high beta) data for response inhibition (Go1-Context1 versus Stim1-Context1) and alterations in S-R mappings involving the least frequent stop-signal of relevance (Stim1-Context1 versus Stim1-Context2), while controlling for differences in overall stop-signal relevance across contexts.

### Unimodal fMRI Analysis

**Figure 4:**
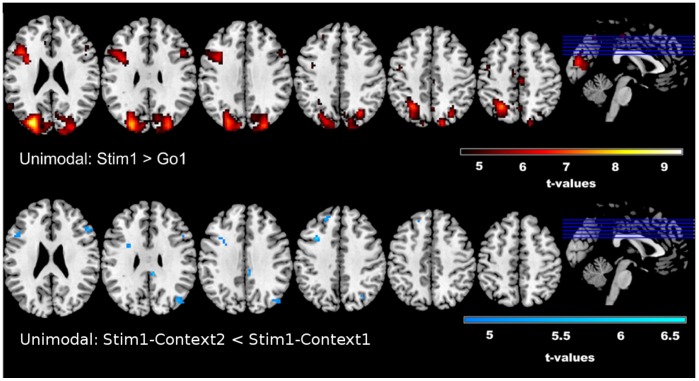
Unimodal fMRI Results. Results from the unimodal fMRI analysis reveal increased activity in the rIFG and left MFG during response inhibition (Stim1> Go) and decreased activity in the rIFG under stimulus-response scenarios in which the most infrequent stimuli demanded inhibition (Stim1-Context1> Stim1-Context2). T-values are reported in Table 2, along with voxel data and MNI co-ordinates.

Dependent samples planned comparisons (t-tests) were computed on the unimodal fMRI (results displayed in Figure 4) data for the two comparisons of interest. Reactive control of response inhibition (seen in Figure 4, top row), as defined by contrasting Stim1-Context1 with Go-Context1 is characterized by increased BOLD signal in the right middle frontal gyrus (MFG) (t = 6.11), extending to the rIFG (t = 5.59). Again, changes in S-R mappings were assessed under two conditions, in which the frequency of the relevant stop stimulus was manipulated. The first contrast for discerning stimulus-related effects involved Stim1-Context1 and Stim1-Context2, where Stim1 constitutes the most infrequently presented stimulus (13%); whereas the second contrast involved Stim2-Context1 and Stim2-Context3, where Stim2 was presented more frequently (26%) than the aforementioned Stim1. Although there were no increased activations associated with either contrast, decreased activity in the rIFG(t = 6.38) is observed during changes to S-R mappings when only the most infrequent stop stimulus (i.e. Stim1-Context1 versus Stim1-Context2) demanded inhibition (Figure 4, bottom row). This reduction in activity was observed for stopping in Context2, where only 13% of stop signals are relevant, as compared to Context1, wherein all stop signals are relevant. This is also congruent with the directionality of the observed unimodal EEG effects. When the contrasts Stim2-Context1 versus Stim2-Context3 is considered, no significant effects in either direction occur. This finding is in correspondence with the unimodal EEG results. See Table 2 for a full list of significantly activated voxels and corresponding t-values for the unimodal fMRI analysis, as well as Tables 3–5 for EEG-informed fMRI analyses with time-frequency parameters. For all tables, locations of maximal activation are reported in MNI co-ordinates. MNI-coordinates and anatomical labelsare provided by the Talairach Atlas daemon [46], [47], freely available at http://www.talairach.org. All effects are significant with a family-wise error (FWE) correction at p<0.05 and with an additional extension threshold of 10 voxels.

**Table 2.Unimodal:** fMRI Results.

Condition	Anatomic Region (BA)	Volume			Coordinates		
		Hemisphere	Voxels	t-max	X	Y	Z
Stim1>Go1							
	Cuneus (19)	Left	2556	9.5	−27	−82	25
	Middle Frontal Gyrus (46)	Left	433	7.26	−42	17	25
	PrecentralGyrus (6)	Right	97	6.66	39	−13	67
	Medial Frontal Gyrus (6)	Right	38	6.39	3	−22	52
	Middle Frontal Gyrus (9)	Right	58	6.11	48	17	31
	Superior Temporal Gyrus (39)	Left	73	5.79	−57	−55	22
	Middle Frontal Gyrus (46)	Right	24	5.59	45	32	16
	Superior frontal Gyrus (8)	Left	11	5.2	−24	41	40
	PostcentralGyrus (3)	Right	11	5.00	18	−40	64
Stim1-Context2< Stim1-Context1							
	Inferior Frontal Gyrus (46)	Right	39	6.38	48	49	16
	Sub-Gyral Frontal Lobe (4)	Right	11	6.28	15	−28	55
	Middle Frontal Gyrus (8)	Left	18	6.24	−33	14	40
	Superior Frontal Gyrus (9)	Left	10	5.42	−18	41	37
	Angular Gyrus (39)	Right	19	5.38	42	−73	31
	Middle Frontal Gyrus (36)	Left	22	5.07	−48	20	22

**Table 3:** Multimodal Results for fMRI Parameterization with Frontocentral (FC) Delta for contrast Stim1> Go1.

Anatomic Region (BA)	Volume			Coordinates		
	Hemisphere	Voxels	t-max	X	Y	Z
Occipital Gyrus (19)	Left	4041	11.82	−36	−82	19
Middle Frontal Gyrus (9)	Left	610	8.80	−45	20	28
Superior Temporal Gyrus (39)	Left	288	7.56	−54	−55	25
Middle Frontal Gyrus (9)	Right	180	6.52	48	14	34
Middle Frontal Gyrus(6)	Left	118	6.16	−33	−1	61
Superior Frontal Gyrus (9)	Right	152	6.52	30	62	28
Middle Frontal Gyrus (10)	Left	125	6.01	−30	65	13
Superior Frontal Gyrus (6)	Left	53	5.91	−3	11	52
Inferior frontal Gyrus (47)	Left	19	5.59	−33	20	−5
Medial Frontal Gyrus (9)	Left	16	5.47	−6	32	31
Superior Temporal Gyrus (39)	Right	20	5.36	51	−61	19
Middle Frontal Gyrus (6)	Right	28	5.35	33	5	64
Cingulate Gyrus (31)	Left	11	5.33	0	−37	28

**Table 4:** Multimodal Results for fMRI Parameterization with Frontocentral (FC) Theta for contrast Stim1> Go1.

Anatomic Region (BA)	Volume			Coordinates		
	Hemisphere	Voxels	t−max	X	Y	Z
Precuneus (19)	Left	3738	10.96	−27	−76	37
Middle Frontal Gyrus (9)	Left	547	8.87	−39	11	31
SupramarginalGyrus (40)	Left	90	6.84	−57	−55	31
Superior frontal Gyrus (10)	Right	137	6.84	18	65	7
Superior Frontal Gyrus(6)	Left	82	6.70	−27	−4	67
Cingulate Gyrus (32)	Left	100	6.53	0	14	41
Middle Frontal Gyrus (9)	Right	107	6.25	45	17	31
Middle Frontal Gyrus (10)	Left	84	5.73	−30	62	7
Middle Temporal Gyrus (22)	Left	45	5.67	−60	−43	4
Medial Frontal Gyrus (8)	Right	42	5.49	3	29	37
Middle Frontal Gyrus (6)	Right	12	5.27	33	2	61
Cingulate Gyrus (31)	Left	15	5.25	0	−37	25

**Table 5:** Multimodal Results for fMRI Parameterization with Left Motor (LM) Beta for contrast Stim1> Go1.

Anatomic Region (BA)	Volume			Coordinates		
	Hemisphere	Voxels	t−max	X	Y	Z
Middle occipital Gyrus (19)	Left	3006	8.96	−33	−82	19
Middle Frontal Gyrus (9)	Left	445	8.38	−45	11	34
Middle Frontal Gyrus (9)	Right	103	5.99	54	20	37
Superior Frontal Gyrus(10)	Right	86	5.94	18	68	13
SupramarginalGyrus (40)	Left	46	5.74	−57	−55	31
Middle Temporal Gyrus (21)	Left	41	5.56	−57	−34	−5
Cingulate Gyrus (31)	Left	14	5.51	0	−37	28
Middle Frontal Gyrus (6)	Left	22	5.49	−36	−1	61
Superior Frontal Gyrus (6)	Left	48	5.20	−3	14	52
Superior Frontal Gyrus (9)	Left	18	5.31	−30	47	28
Superior Frontal Gyrus (8)	Right	22	5.28	24	29	46

### Multimodal fMRI Analysis: EEG-informed fMRI

**Figure 5:**
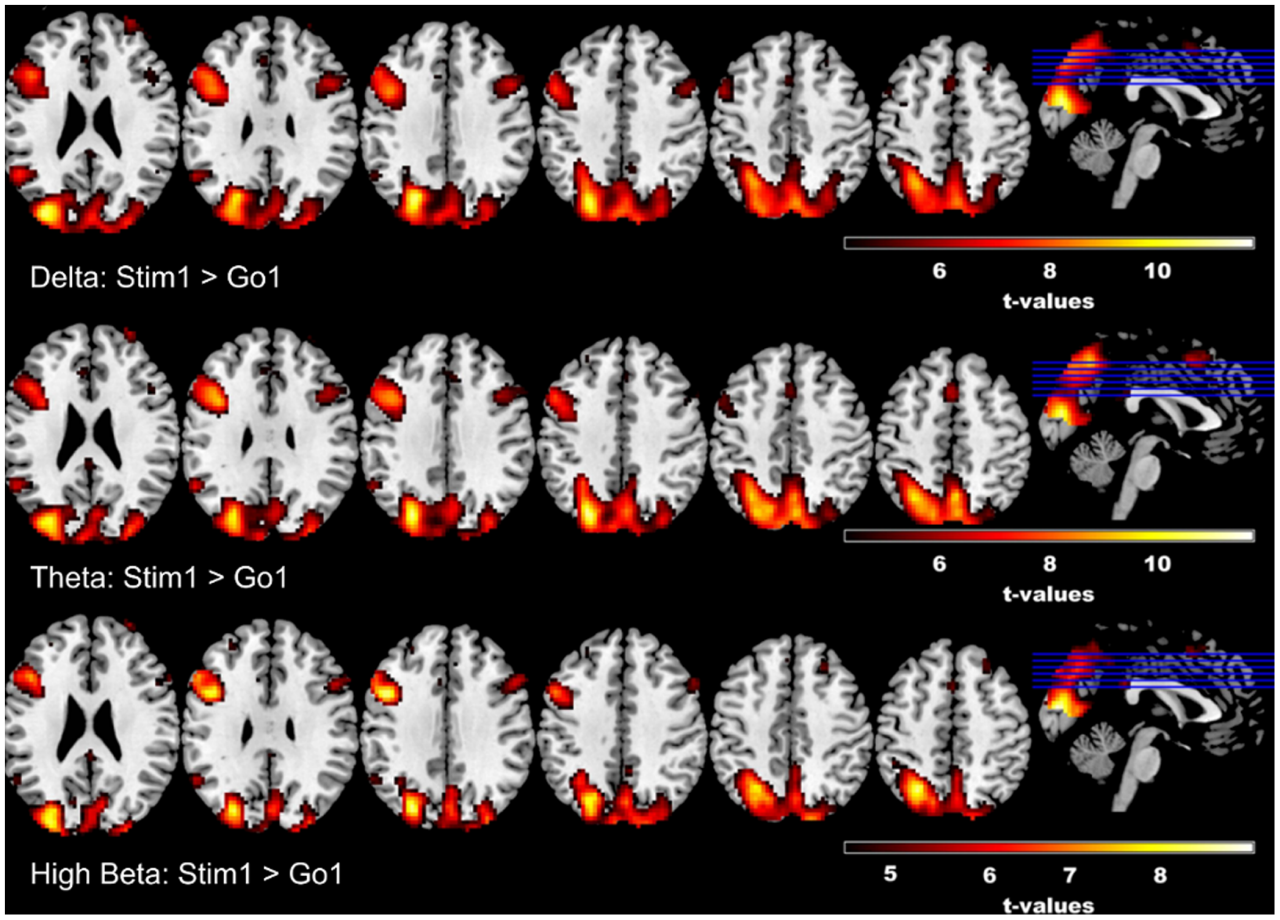
EEG-informed fMRI Results. Results from time-frequency EEG-informed fMRI analyses reveal overlapping effects across frequency bands for response inhibition, especially within the left MFG and to a lesser extent the right inferior frontal region and cingulate gyrus; however, theta band parameterization additionally reveals larger activation within the superior frontal gyrus. T-values are reported in Tables 3–5, along with voxel data and MNI co-ordinates.

The results from the unimodal EEG analyses also guided our EEG-informed fMRI analysis (Figure 5). We chose to parameterize the fMRI BOLD signal with frontocentral delta (Figure 5, top row), frontocentral theta (Figure 5, middle tow), and left motor high beta (Figure 5, bottom row) in three separate second-level flexible factorial designs, based on the observed EEG effects (above). Response inhibition was assessed by comparing Stim1-Context1 with Go-Context1 in the form of paired samples t-tests via SPM8, as this contrast likely reflects the most common comparison used in the stop-signal task – that is, an infrequent stimulus that demands stopping versus the dominant go-signal. Effects of S-R mapping variations were assessed by comparing Stim1-Context1 with Stim1-Context2, as well as Stim2-Context1 with Stim2-Context3; however, most EEG features (aside from high beta) and behavioral measures were significantly modulated by variations to the stop stimuli frequency when the S-R context indicated that only the most infrequent stop signal (i.e. Stim1-Context2) was relevant for stopping. Parameterization of the fMRI with time-frequency data (delta, theta, high beta) revealed a similar pattern of activations for response inhibition that was observed in the unimodal fMRI analysis (See Figure 5) namely common (to all three time-frequency parameters) significantly increased activation patterns in the right IFC, left MFG and cingulate gyrus (See Tables 3–5 for a full list of significantly activated voxels and corresponding t-values). No significant increases or decreases were observed for contrasts testing variations in S-R mappings.

Note, however, that parameterization of fMRI with component activities derived via group ICA [53] revealed associations of EEG and fMRI signals in context of varying S-R mappings (see Figure S1 in File S1). An independent component (IC; See IC 5 in Figure S2 in File S1), characterized by an anterior-posterior topography with predominant low (i.e. delta/theta) time-frequency amplitudes occurring around the latency of the P300, was associated with decreased BOLD activation in frontal regions when the most infrequent (13%) stimuli was relevant (See bottom row in Figure S1 in File S1), as compared to when all stimuli were relevant for stopping (i.e. Stim1-Context1 versus Stim1-Context2). Refer to Table S1 in File S1 for a list of voxels and corresponding t-values for the IC-informed fMRI analysis.

## Discussion

By implementing a modified version of the stop-signal task, including three contextual stimulus-response assignments, we were able to assess functional brain networks and spatiotemporal EEG features characterizing typical response inhibition and how relevant brain mechanisms change with variations in S-R mappings (i.e. altering the relevance of stimuli for stopping). This was accomplished by means of concurrent EEG-fMRI measurements and single-trial EEG- as well as IC-informed (see File S1) fMRI analyses. Behavioral results demonstrated a performance enhancement during the most infrequent stopping condition (Stim1-Context2), where faster goRTs and fewer errors were observed, as compared to other conditions. Such a pattern of results, which cannot be merely explained by a speed-accuracy tradeoff, can arise despite a dynamic tracking procedure given that the SOA was tracked separately for each context. Thus, differences in context-wise stopping accuracy may arise from different performance-related strategies under the three stimulus-response mapping contexts employed by the subjects. Unimodal EEG analyses indicated that frontocentral and centroparietal delta and theta activity increased during response inhibition, as did bilateral motor low-beta and bilateral frontal high-beta activity. Increased activations in the rIFC and left MFG were observed for response inhibition. Multimodal analyses via EEG-informed fMRI demonstrated increased rIFC and left MFG activations with response inhibition to be associated with frontocentral delta and theta as well as left motor high beta. Multimodal, as well as unimodal EEG and fMRI analyses revealed decreased activity in regions (fMRI) and frequency bands (EEG) typically involved in response inhibition when only the most infrequent stimuli demanded inhibition (Context2), as compared to when all deviant stimuli demanded stopping (Context1). These aforementioned EEG and fMRI results, in conjunction with behavioral effects, cannot be explained by a purely stimulus frequency-driven hypothesis and indicate an interaction between task contexts, stimuli-type and stimulus-frequency in the context of inhibition.

Behaviorally, when subjects had to inhibit their response to the most infrequently presented stimulus (Stim1-Context2), performance was enhanced as indicated by faster goRTs and fewer commission errors when compared to the context wherein all deviant stimuli (that is, both Stim1 and Stim2 in Context1) were relevant for stopping. Such effects were not found with Context3, i.e. when the stopping stimulus was presented after 26% of go-stimuli, a pattern congruent with unimodal EEG and fMRI, as well as the multimodal effects. These results suggest a performance benefit (e.g. faster goRT, reduced response rates) that cannot be explained by a simple speed-accuracy tradeoff because when the most infrequent stimulus demands inhibition (that is, Stim1-Context2), subjects commit fewer false alarms (i.e. less commission errors) and demonstrate faster go-RTs in comparison to the other conditions. If behavioral results could be explained by a frequency-driven effect, one would expect elevated error rates with faster go-RTs during conditions when stopping demands were most infrequent; however, our data show a different pattern of results.

Overall, response inhibition was characterized by increased rIFC and left MFG activity, as well as increased delta, theta and beta activity, when compared to go-trials. Correspondingly, multimodal analyses, involving parameterization of the fMRI with EEG time-frequency characteristics revealed a similar pattern of effects for increased left MFG and rIFC BOLD signal activity during stopping when the BOLD signal was parameterized with delta, theta and high-beta band activity.

Contextual stopping of the most infrequent stimulus (i.e. Stim1-Context2) was associated with attenuated increases in activity in the delta, theta and beta bands and in the rIFC when compared to the frequency-matched stimuli wherein the context demanded all deviant stimuli require inhibition (that is, Stim1-Context2< Stim1-Context1). These results demonstrate relative decreases in neurophysiological reactivity (i.e. attenuated increase in EEG and rIFC activity) yet increased behavioral performance (i.e. faster goRTs, fewer errors) under conditions of most infrequent stopping. Reduced activity in the rIFC during infrequent stopping (Stim1-Context2< Stim1-Context1) was observed in the unimodal fMRI analysis as well as in the multimodal analysis involving an independent component (that is, IC5), that was dominated by delta and theta activity, as well as variations in early alpha and beta activity, reflecting a possible interaction between ‘early-selection’ processes and possible performance monitoring. Since the directionality of the effects is in correspondence with both unimodal EEG and fMRI analyses, these results would elude to the efficacy of using additional data decomposition techniques (i.e. group ICA) for combined multimodal analyses [53], [54].

The aforementioned neurophysiological effects observed in the context demanding the response to the most infrequent stimuli to be withheld, suggest more efficient cognitive processing during the most infrequent stopping condition, which may possibly be mediated by the rIFC, due to its role in response inhibition and attentional control [48]. Given that the rIFC is specifically involved in a response inhibition related network [1], and that delta and theta band activity have been implicated in cognitive control processes contributing to successful inhibition [26], these findings are in accordance with studies suggesting a neural efficiency hypothesis [49]. Such neurophysiological and behavioral evidence of neural efficiency during variations of S-R mappings within the stop-signal task have not yet been reported in the literature and may have applications extending into the clinical domain, as we will discuss later. Furthermore, activation in the cingulate gyrus during response inhibition was observed when theta-frequency parameterization was utilized, which is congruent with findings demonstrating the role of the cingulate cortex in cognitive control [50].

Cognitive efficiency theories would suggest that when some cognitive operations are performed quickly, resource allocation within specific neuronal networks could be minimized and performance would be maximized [49].The behavioral evidence for cognitive efficiency explanations originates from processing speed in cognitive tasks [49]; however, our results extend beyond purely behavioral processes. EEG data subjected to a time-frequency analysis revealed regional decreases in delta and theta activity, spanning both frontocentral and centroparietal EOIs under conditions of infrequent stopping, as compared to when all infrequent stimuli are relevant for inhibition. Due to the close concordance between delta and theta activity with specific cognitive processes related to response inhibition such as conflict monitoring and evaluation [26],we suspect that these results reflect an electroencephalographic representation of neural efficiency under relatively infrequent stopping demands, for conditions involving multiple stimuli. Unimodal fMRI data also demonstrate a relative decrease in BOLD signal in the rIFG (right inferior frontal gyrus, pars triangularis) for this contrast, which has previously been implicated in an inhibition-related network [51]. Through which mechanism such neural efficiency is achieved requires further investigation. One may hypothesize that it is due to an active preparation of a stopping network for ‘proactive’ control under contexts where the most infrequent stimuli demand inhibition, which is otherwise not prepared during normal response inhibition wherein all stimuli are relevant for stopping [19]. That is, in the second context, the infrequent stimulus indicating that stopping is required, may elicit more attentional mechanisms due to foreknowledge of the stopping demands, based on the current context. Therefore, the relative amount of recruitment from the stopping network, including the rIFC, is comparatively smaller when only a subset of stimuli are relevant, as compared to when all stimuli are relevant. Since proactive control can also be conceptualized as a form of actively maintaining current goals in a sustained manner [4], attentional and perceptual systems might be optimally biased, which could translate to priming or preparing the specific neural network resulting in improved (i.e. more efficient) neurocognitive processing.

Although the results of this study have provided an avenue for understanding the interaction between stimulus-response contingency and response inhibition processes, the implications may be more prudent when considering impaired response inhibition in clinical disorders. The results reported within this study conducted on healthy participants suggest that behavioral inhibition may be more multi-faceted than originally thought, as the results were not in accordance with a purely stimulus frequency-driven explanation. If the aforementioned neural efficiency explanation holds true in clinical populations, then these results would certainly change the interpretation of many studies reporting impaired response inhibition in clinical disorders such as schizophrenia [6]–[9], [52], AHDH [10], [11] and OCD [12]. If response inhibition is prone to influence from other cognitive functions, such as those used during updating and maintaining goals during changes to S-R mappings, then it would be necessary to uncover how patients perform in such tasks to further disentangle cognitive impairments. This would carry profound implications for not only a more thorough understanding of dysfunctional cognitive processes, but also for cognitive and/or behavior-focused treatments for patients.

## Conclusion

An interaction of altered S-R mappings and the frequency of relevant stop stimuli was revealed, showing that inhibition to infrequent stimuli in the context of task-irrelevant stimuli is characterized by a relative decrease of activity in regions, such as the rIFC, typically involved with reactive control of response inhibition. This would suggest a type of neural efficiency, which takes place when subjects have foreknowledge about which stimuli demand attention. These results, when taken in to account with the behavioral findings, would suggest a facilitative role of infrequent stopping demands during the presence of multiple stimuli, demonstrating an interaction between both foreknowledge and stimulus frequency. Utilizing information from independent components for parameterized fMRI analysis revealed an association of a primarily delta/theta dominant independent component with a decrease in rIFC activity that was otherwise not observed for the time-frequency data. These results not only provide further evidence for the utility of performing a decomposition of EEG data when conducting further multimodal EEG-fMRI analysis, but also provide a new perspective for interpreting results from response inhibition studies that manipulate S-R mappings. Further investigations to discern interactions of S-R mappings and stop-signal frequencies within clinical populations are warranted, in order to develop a richer model of response inhibition during contextual stopping.

## Supporting Information

File S1 **Supporting Information on Decomposition of Multi-Subject EEG Data and group-level ICA.** The steps involved in the decomposition of multi-subject EEG data are outlined, with additional information provided on the IC-informed fMRI parameterization and the subsequent multimodal IC-informed fMRI analysis. Table S1 contains multimodal results from the IC-informed fMRI analysis. Figure S1. IC-informed fMRI Results. Results from the IC EEG-informed fMRI analyses illustrate that IC3 and, to some extent, IC5 demonstrate regional increases in the left MFG for response inhibition that resemble results presented for the unimodal and time-frequency EEG informed fMRI analysis Figure 4 and 5, respectively). The inhibition control network depicted in IC1 implicates posterior regions. IC1 and IC5 both demonstrate significant regional decreases under stimulus-response mappings requiring infrequent stopping. T-values are reported in Table S1 in File S1, along with voxel data and MNI co-ordinates. Figure S2. Independent Component time and frequency characteristics. Component topographies for IC1 (left) to IC5 (right) are shown along with the corresponding component time-frequency plots, showing bootstrapped power values for all stimuli (first row), go-stimuli (second row), as well as Stim1 (third row) and Stim2 (fourth row) averaged across all three contexts. Figure S3. Low Beta time frequency plot. Low beta power across contexts and stimuli is presented for reasons of consistency, as the low beta plot (see Figure 3) only displays power across EOIs rather than across contexts.
(DOCX)
