# Supplementary material for: Stimulus-Response Mappings Shape Inhibition Processes: A Combined EEG-fMRI Study of Contextual Stopping
Source: PLoS One. 2014 Apr 24;9(4):e96159. doi: 10.1371/journal.pone.0096159 (PMC3999100; doi:10.1371/journal.pone.0096159)
Supplement: File S1 — Supporting Information on Decomposition of Multi-Subject EEG Data and group-level ICA. The steps involved in the decomposition of multi-subject EEG data are outlined, with additional information provided on the IC-informed fMRI parameterization and the subsequent multimodal IC-informed fMRI analysis. Table S1 contains multimodal results from the IC-informed fMRI analysis. Figure S1. IC-informed fMRI Results. Results from the IC EEG-informed fMRI analyses illustrate that IC3 and, to some extent, IC5 demonstrate regional increases in the left MFG for response inhibition that resemble results presented for the unimodal and time-frequency EEG informed fMRI analysis Figure 4 and 5, respectively). The inhibition control network depicted in IC1 implicates posterior regions. IC1 and IC5 both demonstrate significant regional decreases under stimulus-response mappings requiring infrequent stopping. T-values are reported in Table S1 in File S1, along with voxel data and MNI co-ordinates. Figure S2. Independent Component time and frequency characteristics. Component topographies for IC1 (left) to IC5 (right) are shown along with the corresponding component time-frequency plots, showing bootstrapped power values for all stimuli (first row), go-stimuli (second row), as well as Stim1 (third row) and Stim2 (fourth row) averaged across all three contexts. Figure S3. Low Beta time frequency plot. Low beta power across contexts and stimuli is presented for reasons of consistency, as the low beta plot (see Figure 3) only displays power across EOIs rather than across contexts. (DOCX) [file pone.0096159.s001.docx]

*Decomposition of Multi-Subject EEG Data*

EEG recordings at specific electrode sites on the scalp will usually reflect multiple processes concurrently. This multidimensional overlap (in the frequency, temporal, and spatial domains), intensified further through volume conduction, can pose a problem when one is interested in deriving EEG measures for a specific neural or cognitive process only. Several methods for the decomposition of EEG data exist, of which blind source separation via independent component analysis (ICA) is an increasingly popular one. Temporal ICA, which is the most common ICA application to EEG, models the multi-channel recordings as linear combination of topographies and time courses while maximizing the statistical independence of the latter. Although basic models are restricted to single subject data, newer developments allow for the concurrent analysis of multi-subject, multi-channel data sets, thereby allowing for straightforward inferences at the group level.
 Here, a group ICA of multi-subject EEG data was computed using EEGIFT[53]. Group ICA extracts statistically independent components consistently expressed across participants. In short, each single-subject dataset first undergoes an individual principal component analysis (PCA), thereby extracting the most relevant and orthogonal time courses. These first-level principal components are then used as variables in a second-, group-level PCA, which estimates the most relevant and uncorrelated principal component time courses across subjects. Ultimately, these time courses are subjected to an ICA that finally computes the statistically independent component time courses. Importantly, this nested procedure is capable of capturing some of the topographical variability typically observed with EEG events found in each of the single-subject datasets of a multi-subject study. Please refer to Eichele et al. (2011), for a more detailed description of the algorithm. For all subjects, trials from the different conditions were randomly chosen and concatenated such that 1) the same number of trials was extracted from a given condition for every subject and 2) that the order of trials with respect to what condition they belonged to was identical across subjects. From this multi-subject dataset five independent components were extracted using group ICA, because two procedures suggested this to correspond to the intrinsic dimensionality of the data. Initially, maximum likelihood estimation was applied to each of the single-subject datasets, already suggesting an average model order of five. The ICASSO software package was used to assess the reliability and stability of the ICA solutions given the extracted number of components based on 100 separate ICA computations. This combination of group ICA and ICASSO[54]was used for evaluating models with 5, 6 and 7 components. Only the five-component solution revealed sufficient reliability and stability. Each group independent component is characterized by its topography and (multi-trial) time course (See Figure S2 in File S1). However, for the parameterization of the fMRI time series from independent components, data from every single trial and subject are needed. Therefore, for every subject the matrices estimated during the data reduction and group ICA steps were extracted and applied to all available data of that subject. This procedure results in individual independent component time courses for every single subject and trial that nonetheless naturally generalize at the group level.
*IC-informed fMRI parameterization*Because we were not able to differentiate among conditions with varying S-R contingencies with parameterization with time-frequency characteristics during the EEG-informed fMRI analysis, a multi-subject IC decomposition was performed.Values extracted from single-subject back projections of group ICs were utilized to inform the fMRI analysis. ICs were chosen based on their ability to differentiate between conditions, based on their root-mean squared (RMS) value; thus, as further explained below, IC1, IC3, IC4 and IC5 were chosen for IC-informed parameterization. The modeling of the IC-RMS values at the single-subject and group levels was completed in the same fashion as it was for the time-frequency data. That is, all IC-RMS parameters were modeled together in one design at the single-subject level, and separate designs were created for each IC-feature at the second-level in a flexible factorial design. Both multimodal analyses (EEG- and IC- informed fMRI analyses) were computed in SPM8 by creating a flexible factorial model with CONTEXT (1,2,3) and STIMULI (Go, Stim1, Stim2) and SUBJECT as factors, with additional parameterization from the respective EEG features. Planned comparison with the use of repeated measures t-tests were performed to discern multimodal differences for variations in S-R mappings (i.e. Stim1-Context1 vs. Stim1-Context2) and reactive response inhibition (Stim1-Context1 vs. Go-Context1) in both directions (i.e. increases and decreases). These contrasts were chosen to further elucidate multimodal characteristics of observed behavioral and corresponding unimodal EEG effects. Hence, planned comparisons were more suitable to test the specific cognitive processes of interest, including what EEG-fMRI features contribute to successful response inhibition and whether or not these effects change under variations in S-R mappings.
*Multimodal fMRI Analysis: IC-informed fMRI* Parameterizations with root-mean squared (RMS) values from ICs calculated from a group ICA were computed to further analyze the data. Only those RMS values which were able to significantly differentiate between stimuli (Go, Stim1, Stim2) across the three contexts at a p<0.05 threshold were further utilized for multimodal parameterization. Figure S2 in File 3 depicts the five resultant IC topographies and the corresponding time-frequency plots after bootstrapped statistics (p<0.001) were applied. With respect to IC3 and IC5, significantly increased activation in the left MFG is observed for successful response inhibition. This is a similar, albeit more focal, pattern of activation which was observed in the time-frequency EEG-informed fMRI analyses. Furthermore, IC1 reveals a posterior network associated with response inhibition (Figure S1 in File S1). Under varying S-R mappings, no significantly increased activations in the direction of Context1 were observed with a family-wise error correction; however, decreased activation in a region specifically implicated in a response-inhibition network, the rIFC, was observed when IC5 was considered (Figure S1 in File S1). This decrease in activation is congruent with the unimodal EEG time-frequency analysis, where significantly decreased activity was observed for the context wherein the most infrequent stop signal was relevant. Moreover, implication of the rIFC is methodologically relevant because this type of activation was not observed in the EEG-informed fMRI analysis and is also conceptually relevant because the rIFC is involved in inhibition-related networks [4]. Table 5 contains a full list of significant activations and deactivations.

**Table S1.** Multimodal Results for fMRI Parameterization with Independent Components (IC)

| **Parameter** | **Condition** | **Anatomic Region (BA)** | **Volume** |  |  | **Coordinates** | | |
| --- | --- | --- | --- | --- | --- | --- | --- | --- |
|  |  |  | **Hemisphere** | **Voxels** | **t-max** | **X** | **Y** | **Z** |
| IC1 | Stim1 > Go1 | | | | | | | |
|  |  | Middle Occipital Gyrus (18) | Left | 276 | 6.81 | -12 | -88 | 13 |
|  |  | Superior Parietal Lobule (7) | Left | 15 | 5.08 | -27 | -61 | 49 |
|  |  | Precuneus (19) | Left | 15 | 5.01 | -24 | -76 | 34 |
|  | Stim1-Context2 < Stim1-Context1 | | | | | | | |
|  |  | Claustrum (Sub-lobar) | Right | 20 | 5.42 | 27 | -13 | 22 |
|  |  | Middle Frontal Gyrus (6) | Right | 10 | 5.42 | 27 | -1 | 46 |
|  |  | Inferior Parietal Lobule (40) | Left | 10 | 5.13 | -48 | -37 | 40 |
| IC3 | Stim1 > Go1 | | | | | | | |
|  |  | Cuneus (18) | Right | 27 | 5.98 | 15 | -97 | 13 |
|  |  | Middle Frontal Gyrus (9) | Left | 61 | 5.89 | -48 | 20 | 31 |
|  |  | Inferior frontal gyrus (45) | Left | 23 | 5.55 | -54 | 26 | 16 |
|  |  | Precuneus (19) | Left | 55 | 5.48 | -27 | -76 | 43 |
|  |  | Middle Occipital Gyrus (19) | Left | 32 | 5.42 | -30 | -85 | 10 |
|  |  | Cuneus (18) | Right | 34 | 5.26 | 3 | -91 | 10 |
| IC4 | Stim1 > Go1 | | | | | | | |
|  |  | Cuneus (19) | Left | 21 | 5.42 | -27 | -79 | 31 |
|  |  | Posterior Cingulate (30) | Right | 10 | 5.10 | 15 | -64 | 7 |
| IC5 | Stim1 > Go1 | | | | | | | |
|  |  | Cuneus (18) | Right | 51 | 5.76 | 3 | -91 | 13 |
|  |  | Middle Frontal Gyrus (46) | Left | 20 | 5.61 | -48 | 20 | 25 |
|  |  | Middle Occipital Gyrus (19) | Left | 18 | 5.16 | -30 | -91 | 22 |
|  | Stim1-Context2 < Stim1-Context1 | | | | | | | |
|  |  | Middle Frontal Gyrus (9) | Right | 11 | 5.96 | 36 | 35 | 25 |

**Figure S1**


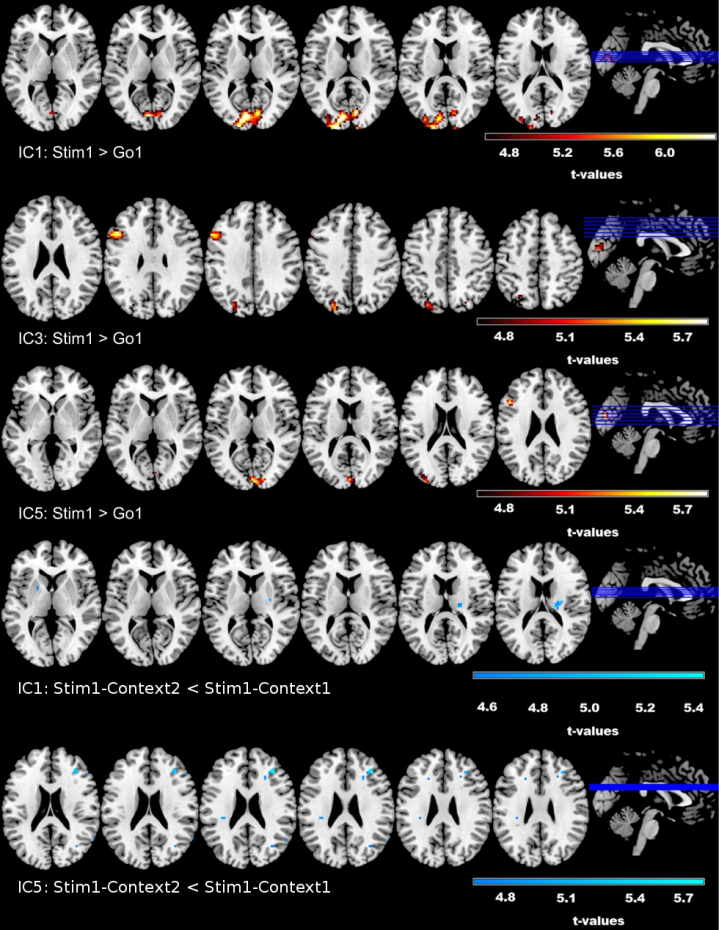


**Figure S2**


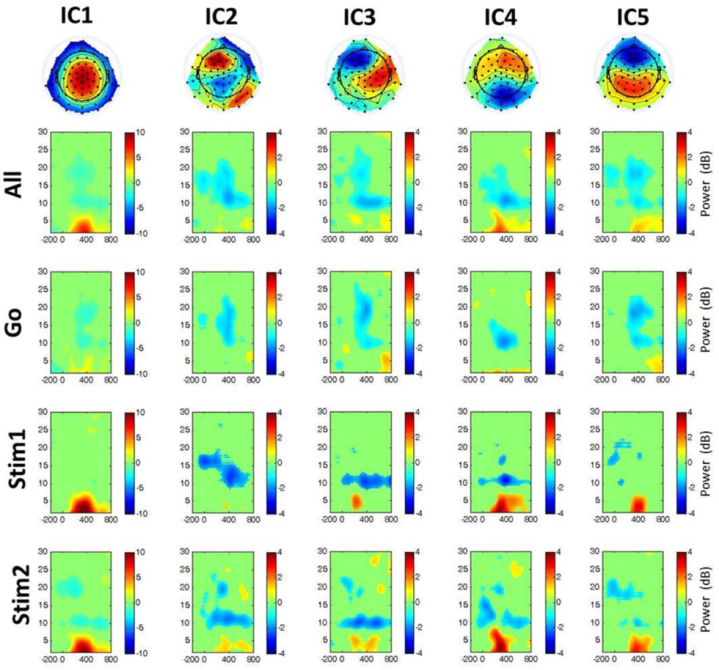


**Figure S3**
